# Comparing open conventional carpal tunnel release with mini-incision technique in the treatment of carpal tunnel syndrome: A non-randomized clinical trial

**DOI:** 10.1016/j.amsu.2020.05.001

**Published:** 2020-05-16

**Authors:** Jalaluddin Khoshnevis, Hojjat Layegh, Negin Yavari, Gita Eslami, Abolfazl Afsharfard, Seyed Mohammad Reza Kalantar-Motamedi, Sina Zarrintan

**Affiliations:** a Division of Vascular & Endovascular Surgery, Department of Surgery, Shohada-Tajrish Medical Center, Shahid Beheshti University of Medical Sciences, Tehran, Iran; b Department of General Surgery, Ardebil University of Medical Sciences, Ardebil, Iran; c Research Department, Tehran Heart Center, Tehran University of Medical Sciences, Tehran, Iran; d Department of Microbiology, School of Medicine, Shahid Beheshti University of Medical Sciences, Tehran, Iran; e Cardiovascular Research Center, Tabriz University of Medical Sciences, Tabriz, Iran

**Keywords:** Carpal tunnel syndrome, Open carpal tunnel release, Mini-incision carpal tunnel release

## Abstract

**Background:**

Carpal tunnel syndrome (CTS) is the most common nerve entrapment neuropathy which is the result of the compression of the median nerve in the wrist. Currently, there is no consensus about the best treatment option. The purpose of this clinical trial was to compare the clinical outcomes of patients undergoing open CT release with mini-incision CT release.

**Patients and methods:**

This clinical trial included 75 patients with CTS who were divided into two groups of 45 and 30 patients to undergo open-CT release or mini incision CT release respectively. Patients were evaluated pre-operatively, days after the surgery and then five months after the operation to record outcomes. At follow-up, the visual analogue scale (VAS) scores for pain, patients’ satisfaction, return to work, length of scar, paresthesia, grip and opposition strength were measured.

**Results:**

A total of 75 patients (mean age: 52.13 years, 73.3% female) underwent CTS surgery. Forty-five patients (60%) had open-CT release and 30 patients (40%) had mini-incision CT release. Postoperative pain and scar length were significantly lower in the mini incision group compared to open group (p < 0.001). The mini-incision CT group returned to work earlier than open group with higher satisfaction (p < 0.001). No significant differences were observed between two groups in respect to the improvement of the opposition, grip and paresthesia (p > 0.05).

**Conclusion:**

Our study demonstrated that mini-incision CT release improves pain more effectively and has better quality of life because of smaller length of scar, immediate return to work and higher overall satisfaction. Neurosensory and motor improvements were also seen in both techniques with the same clinical impact.

## Highlights

• Mini-incision release in CTS improves pain and patients have better quality of life.
• Mini-incision release in CTS has smaller length of scar.
• Mini-incision release in CTS is associated with earlier return to work compared to conventional technique.

## Credit author statement

Jalaluddin Khoshnevis: Conceptualization, Data curation, Funding acquisition, Investigation, Methodology, Project administration, Resources, Supervision, Validation, Visualization, Writing - review & editing. Hojjat Layegh: Data curation, Formal analysis, Funding acquisition, Investigation, Methodology. Negin Yavari: Data curation, Formal analysis, Software, Writing - original draft, Writing – review & editing. Gita Eslami: Formal analysis, Validation. Abolfazl Afsharfard: Conceptualization, Data curation, Methodology, Validation . Seyed Mohammad Reza Kalantar-Motamedi: Conceptualization, Methodology, Validation, Visualization. Sina Zarrintan: Conceptualization, Data curation, Formal analysis, Software, Writing - original draft, Writing - review & editing.

## 1 Introduction

Carpal tunnel syndrome (CTS) is one of the most prevalent forms of nerve entrapment neuropathy worldwide [1]. The carpal tunnel (CT) is a fibro-osseous tunnel in the volar part of the wrist. It extends from the wrist flexion crease to the distal border of the thenar eminence and it contains median nerve and the tendons of the flexor digitorum superficialis, flexor digitorum profundus and flexor pollicis longus. CTS is a condition due to compression of the median nerve as it traverses through carpal tunnel. Diagnostic criteria of CTS are nocturnal numbness, numbness and tingling in the median nerve distribution, weakness and/or atrophy of thenar muscles together with positive Tinel's sign, painful Phalen's maneuver and weakened opposition [2,3]. There are variety of potential risk factors to develop CTS including gender, obesity, old age, pregnancy, work activities demanding frequent use of force and vibratory tools [[4], [5], [6]], and concurrent medical conditions such as diabetes mellitus, rheumatoid arthritis and thyroid disorders [[7], [8], [9], [10]].

The high prevalence of CTS and its effect on quality of life makes it worthy of evaluation to find an effective method of treatment which would be cost-effective and satisfactory [1]. Non-surgical modalities are mostly preferred in mild to moderate stages. These include wrist splints or corticosteroid injections. Surgical treatment is generally performed for severe cases with persistent clinical symptoms [3,11]. Various techniques have been used including open-CT release and mini-incision CT release [12], but there is still lack of evidence regarding the best technique that provides better outcomes. In this study, we aimed to make a comparison between the outcomes of open-CT release and mini-incision CT release in patients with CTS.

## 2 Patients and Methods

### 2.1 Study population and variables

**Fig. 1:**
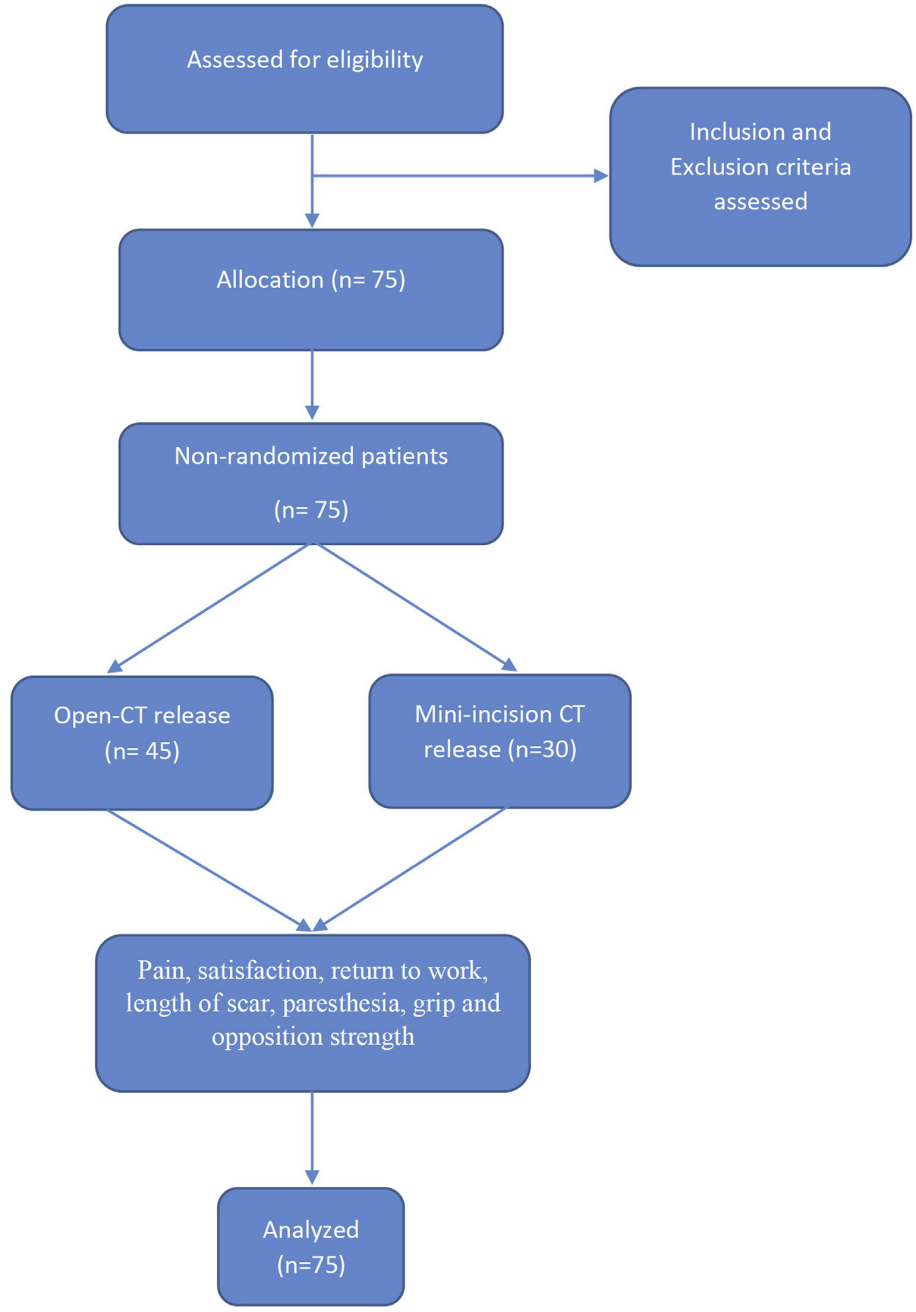
Flowchart of patients in two groups of open and mini-incision carpal tunnel release.

The study was a non-Randomized Clinical Trial conducted prospectively on 75 patients who had carpal tunnel syndrome. The patients were admitted to Shohada-Tajrish Medical Center, Shahid Beheshti University of Medical Sciences, Tehran, Iran. Initially, diagnosis was made by physical examination findings. The findings were numbness in the distribution of the median nerve, nocturnal paresthesia, weakness and atrophy of the thenar muscles, presence of a positive Tinel's sign and/or painful Phalen's maneuver. Electrodiagnostic confirmation of the diagnosis was also performed by electromyography (EMG) and Nerve Conduction Velocity (NCV) studies. EMG-NCV results were in favor of moderate to severe CTS in all patients. Moreover, exclusion of other peripheral neuropathies was done. The inclusion criteria were having moderate to severe CTS, informed consent of the patient to undergo surgical treatment and electrodiagnostic confirmation of the CTS. Exclusion criteria were presence of other peripheral neuropathies of the upper extremities, steroid injection at carpal tunnel within two months prior to surgery, recent carpal tunnel surgery at the contralateral hand within six months prior to current hospitalization and ipsilateral thoracic outlet syndrome. Study variables consisted of demographic data, treatment option, time of return to work, post-operative length of scar, post-operative pain, satisfaction and alleviation of signs and symptoms. Patients were divided into two groups: 1) Forty-five patients who underwent open CT release, 2) Thirty patients who underwent mini-incision CT release. The entire patients were followed up for one year. This work is being reported in line with Consolidated Standards of Reporting Trials (CONSORT) Guidelines [13]. However, this study was a non-randomized clinical trial and was performed as a quasi-experimental study. Fig. 1 illustrates the study flowchart. Clinical outcomes included improvements in grasp, opposition and paresthesia. The motor tests of measurements of hand grip and opposition strength were performed according to standard procedures using a dynamometer and a pinch meter. Digital sensibility of the thumb, index, and middle fingers was measured using Semmes-Weinstein monofilaments. The postoperative pain was assessed by visual analogue scale (VAS) scores. Patient-reported outcomes were evaluated using a questionnaire. Patients reported a visual analogue scale (VAS) score for pain at rest and during activity. The VAS is a horizontal 100-mm line with a scale from 0 to 10 (0 equaling no pain and 10 corresponding to the most intense pain). Patients were asked to grade their pain by drawing a stripe along this line. Overall satisfaction of patients was also evaluated in the same manner. The reported satisfactions and dissatisfactions were demonstrated by excellent, good, fair, poor and very poor feelings. The patients were recruited from September 16, 2015 to June 21, 2016 and the sample size was estimated by the volume of patients at our center during the trial period.

### 2.2 Operative technique

The patients in group one underwent open CT release under local anesthesia while using an upper-arm tourniquet. A skin incision was made extending from Kaplan's cardinal line distally to beyond the wrist crease proximally. The transverse carpal ligament was reached through the elimination of the subcutaneous fatty tissue and palmar fascia while avoiding damage to superficial palmar branch of the median nerve. A dissection was then made and all the component of the carpal tunnel was released. The patients in group two underwent mini-incision CT release under local anesthesia with lidocaine injection. A 1-cm incision was marked in the palm, beginning at the intersection of Kaplan's cardinal line and a line drawn along the radial border of the third web. A blunt Metzenbaum scissor was used to release the medial nerve within the carpal tunnel through the mini-incision. All the operations were performed at the department of surgery, Shohada-Tajrish Medical Center, Shahid Beheshti University of Medical Sciences, Tehran, Iran. Both methods of operations were performed by three surgeons who were experts in hand surgery and carpal tunnel release. The surgeons have been working as attending physicians in a high volume center of trauma, vascular and hand surgery for decades and have sufficient clinical expertise. They also used routine perioperative care for patients of both groups. Also, all measures were the same for patients of two groups except the type of the surgery. Likewise, the investigator of clinical outcomes was not aware of the type of operations. Thus, there were potential quality control measures to assure standardization of the procedures and postoperative evaluations.

### 2.3 Statistical analysis

All data of patients were submitted to a checklist and were analyzed. Statistical analysis was conducted by SPSS 21.0 software. Continuous and categorical variables were described by mean ± standard variation (SD) and frequency (%) respectively. In quantitative variables, we used Student *t*-test for normally distributed data. We assessed the association between qualitative variables using chi square or Fisher's exact tests. P values less than 0.05 was considered statistically significant. The protocol of this study was approved by research deputy of Faculty of Medicine, and Research Vice-Chancellor office of Shahid Beheshti University of Medical Sciences, Tehran, Iran. Informed consents were obtained by surgical residents and attending physicians from all patients prior to their enrolment to the study. The protocol of this study was registered to Iranian Registry of Clinical Trial (IRCT) and was approved by the number *IRCT20191019045155N3*.

## 3 Results

### 3.1 Population

All 75 patients (mean age: 52.13 years, 73.3% female) who underwent CTS surgery at our center were successfully followed and were included in the final analysis. Demographic data of the study population are shown in Table 1. Baseline demographics did not have significant differences in two groups. Diabetes mellitus or thyroid disorders were not present in any of the patients of two groups.

**Table 1:** Baseline characteristics and outcome variables in the study population.

Variables	Open CT release (n = 45)	Mini-incision CT release (n = 30)	P-value
Age (Years), mean (SD)	54.47 (13.88)	49.90 (14.31)	>0.05
Gender	Male: 11 (24.4) Female: 34 (75.6)	Male: 9 (30.0) Female: 21 (70.0)	>0.05
Hypertension	6 (13.3)	2 (6.7)	>0.05
Grasp-grip improvement, n (%)	39 (86.7)	27 (90.0)	>0.05
Opposition improvement, n (%)	39 (86.7)	27 (90.0)	>0.05
Scar^a^, mean (SD)	4.18 (0.58)	1.70 (0.33)	<0.001
Paresthesia improvement, n (%)	37 (82.2)	25 (83.3)	>0.05
Pain, mean (SD)^b^	7.09 (0.97)	1.83 (0.87)	<0.001
Return to work, mean (SD)^c^	24.07 (4.99)	9.37 (2.55)	<0.001
Satisfaction, n (%)			<0.001
Excellent	6 (13.3)	21 (70.0)	
Fair	15 (33.3)	8 (26.7)	
Good	15 (33.3)	1 (3.3)	
Poor	5 (11.1)	–	
Very poor	4 (8.9)	–	

Continuous variables are presented as mean (SD); Categorical variables are described as frequency (percentage); n (%); CP – Carpal Tunnel.

a Centimeters (cm).

b Measured by Visual Analogue Scale (VAS).

c Days.

### 3.2 Clinical outcomes

Length of scar was significantly smaller in mini-incision technique (p < 0.001). Post-operative pain was significantly lower in mini-incision group (p < 0.001). Return to work and satisfaction were also significantly faster and higher in mini-incision group compared to open-CT release respectively (both p-values were <0.001). There were no differences in improvements of opposition, grip and paresthesia in two groups (p > 0.05). There were no wound infection or nerve damages in patients of either open or mini-incision groups. Table 1 illustrates the clinical outcomes of the patients of two groups.

## 4 Discussion

Carpal tunnel syndrome is one of the most common neuropathies affecting peripheral nerves of upper limbs. Carpal tunnel decompression surgery has evolved over the years to decrease the complications associated with the surgical procedures. The standard open CT release has been the optimal procedure for surgical decompression of the median nerve. Although this technique has the advantage of direct visualization of the structures, it may be associated with certain complications such as a painful scar, neurosensory deficits and neuromas. The complications can decrease the strength of the hand and quality of life. Even though the mini-incision CT release have limited visualization, it is not yet proved to have increased rate of complications [14].

Our study revealed that length of scar, pain, return to work and satisfaction are significantly improved in mini-incision CT release compared to open CT release. Paresthesia, grip strength and opposition did not have any differences in two study groups and they were improved equally. A prospective study also showed that cosmetic and functional results are significantly improved in mini-incision CT release and thus the technique is safe and useful [15]. In another study, Cowan et al. investigated 65 patients with limited incision open CT release. They demonstrated that patients returned to modified and full duties on averages of 11.8 and 18.9 days after the surgery respectively [16]. However, in our study, the average return to work was 24.06 ± 4.99 and 9.37 ± 2.55 days in open CT release and mini-incision CT release groups respectively. Moreover, Tzaan and colleagues performed mid-palmar accurate incision on 84 patients with CTS. A total of 78 hands (81%) had excellent relief of symptoms such as numbness, pain and muscle weakness. The average return to work was 4.5 weeks. Postoperative pain was noted in seven hands (7%) [17].

In a study conducted by Keramettin and colleagues, they performed open CT release on 73 patients and mini-incision release in 56 patients. They showed that grip strength, pinch improvement and cosmetic results were better in mini uni-skin incision compared to open CT release [18]. In addition, Iida et al. evaluated minimum incision open CT release on 108 patients. Nocturnal or daytime dysesthesia were completely relieved in 94% of patients. No painful or hypertrophic scar formation was observed in the patients [19]. Çokluk et al. also performed double mini skin incision on 24 female patients. They observed reduced scar formation on the palmar surface of the hand with this technique [20]. In addition, Elsharif et al. conducted a study on 43 and 39 patients who underwent open release and minimally-invasive Knifelight method respectively. Their results showed that the minimally invasive Knifelight carpal tunnel release was associated with better clinical outcomes in respect to pain release, recurrence or scar-related problems and a high level of general satisfaction [21]. Our findings showed that paresthesia, grip strength and opposition were not different in two groups. This suggests that mini-incision technique is not inferior to the conventional open surgery. Furthermore, the mean length of scar in open CT release was 4.18 ± 0.58 cm and it was 1.7 ± 0.34 cm in mini-incision CT release which shows higher rate of success in mini-incision CT release.

In two other studies, there was statistically significant improvement in grip and pinch strengths, and sensory test results in limited incision surgery compared to open CT release. The pain was also lower in mini-incision CT release. Consistent with our study, paresthesia and opposition were equally improved in both types of surgeries [14,22]. Postoperative pain severity was also lower in mini-incision CT release compared to open CT release (VAS: 1.83 ± 0.87 vs. 7.09 ± 0.97) in our series. It can also be understood that neurosensory improvement was also seen in our patients who underwent mini-incision CT release. However, our study was a quasi-experimental trial because randomization was not performed. In addition, the sample size was estimated based on the volume of patients at our center during the trial and it was another limitation of our study.

Based on the results, the outcomes of this study reflect the effectiveness of mini-open CT release. Patients in the mini-incision group had more satisfaction, returned to work earlier, experienced less pain and had smaller length of scar. Paresthesia, grip and opposition strength did not differ in both groups but they improved clinically the same. Therefore, we recommend this technique in CTS for higher quality of life and better outcomes.

## 5 Ethical statement

The protocol of this study was approved by Shohada-Tajrish Medical Center, Shahid Beheshti University of Medical Sciences, Tehran, Iran. The protocol of this study was registered to Iranian Registry of Clinical Trial and approved by the number ***IRCT20191019045155N3**.* The URL is https://www.irct.ir/trial/45380.

## Data statement

Data is available upon request.

## Funding

Shahid Beheshti University of Medical Sciences, Tehran, Iran.

## Provenance and peer review

Not commissioned, Editor review. Peer review was conducted in International Journal of Surgery. The article was transfered here after peer review and acceptance for publication in sister journal "Annals of Medicine and Surgery".

## Declaration of competing interest

The authors do not have any conflicts of interest.
